# Fluorescent and Catalytic Properties of a 2D Lamellar Zn Metal–Organic Framework with sql Network Structure

**DOI:** 10.3390/molecules28176357

**Published:** 2023-08-30

**Authors:** Chaewon Shin, Jongseo Kim, Seong Huh

**Affiliations:** Department of Chemistry and Protein Research Center for Bio-Industry, Hankuk University of Foreign Studies, Yongin 17035, Republic of Korea

**Keywords:** metal–organic framework, lamellar structure, sql network, pyrene, fluorescence, blue-light emission, catalysis, transesterification

## Abstract

A two-dimensional (2D) lamellar Zn metal–organic framework (Zn-MOF, **1**) with a fluorescent 1,6-di(pyridin-3-yl)pyrene (3-DPPy) and 1,4-benzenedicarboxylate (BDC^2−^) bridging linkers was prepared and structurally characterized. The chemical formula of **1** is [Zn(μ-3-DPPy)(μ-BDC)]_n_. The mononuclear Zn(II) ion, acting as a node, is tetrahedrally coordinated with two 3-DPPy and two BDC linkers. The coordination environment of Zn(II) is a distorted tetrahedral geometry. The Zn-MOF is the **sql** network structure based on topology analysis. The undulated 2D sheets of **1** tightly pack together to form a lamellar structure. The pyrene moieties are parallelly oriented to each other. The Zn-MOF is not porous, possibly because the mononuclear Zn(II) node did not form cluster-based secondary building units due to the less symmetric 3-DPPy. The steady-state fluorescence measurements indicate that the fluorescence signal of the **1** is slightly blue-shifted compared to the free 3-DPPy in the solid state. The excimer emission band at 463 nm for crystalline 3-DPPy is shifted to 447 nm for **1**. The value of 447 nm is also a blue-shift value compared to nonsubstituted pyrene crystals (470 nm). Despite its nonporosity, the surface Lewis acidic sites of **1** could catalyze the transesterification of esters. Surface defect sites are responsible for this catalytic activity.

## 1. Introduction

The development of new functional metal–organic frameworks (MOFs) is a flourishing research field due to their versatile framework structures and diverse physicochemical properties, which are apt for a range of applications [1,2,3,4,5,6,7,8]. Selective gas sorption [1,2], guest encapsulation [3,4], and catalytic applications [5,6,7,8] are representative examples of their usage. These important application areas continuously attract many new synthetic attempts for novel functional MOFs aimed at finding better crystalline solid materials. The framework structures of MOFs can be widely and systematically varied on demand because of not only their different multitopic bridging linkers, but also the symmetry of the bridging linkers that interconnect the metal ions. The donor atom types of the bridging linkers are also important for designing the frameworks with certain metal ions because metal ions can preferentially bind certain types of donor atoms [9]. In this regard, dicarboxylate- and tricarboxylate-based bridging linkers are most widely used with metal ions preferentially binding O-donor ligands. MOF-5 [10], MOF-177 [11], and HKUST-1 [12] are representative examples of these types of MOFs. In these cases, no extra bridging ligands other than a dicarboxylate or a tricarboxylate are required for forming porous three-dimensional (3D) frameworks. The formation of cluster-based secondary-building units (SBUs) such as a tetranuclear [Zn_4_O(COO)_6_] SBU for both MOF-5 and MOF-177 and a dinuclear [Cu_2_(COO)_4_] SBU for HKUST-1 is responsible for the efficient construction of 3D frameworks in the absence of other auxiliary linkers. In addition, an In(III) ion can form 3D MOFs with a dicarboxylate without forming cluster-based SBUs. In this case, an eight-coordinate In(III) center coordinated with four bidentate carboxylates acts as a node to effectively form 3D frameworks without any auxiliary bridging linkers [13,14,15].

Additionally, auxiliary N-donor pillar ligands are frequently utilized for constructing MOFs with certain carboxylate-based bridging linkers [16,17,18]. Pillaring by these auxiliary N-donor ligands plays an important role in providing 3D MOFs containing well-defined 2D square grid porosity in a controlled manner. The porosity can be further fine-tuned depending on the physical dimension of the pillar ligands [19,20]. Auxiliary N-donor ligands with linear geometry were commonly employed for the construction of 3D frameworks [21]. Auxiliary N-donor ligands with low-symmetry structures also led to porous 3D frameworks with interesting structures. Meanwhile, the chemical structures of potentially ditopic di(pyridine)-functionalized pyrene derivatives are very versatile. Geometric isomers of di(pyridine)-functionalized pyrenes with different substitution positions such as 1,6- and 2,7-positions were reported [22,23,24], and each isomer can induce distinct extended frameworks. Thus, they are good bridging ligands in forming new framework structures. Additionally, the pyridine-substituted pyrene bridging ligand is interesting because of its large physical dimension and potentially blue light-emitting properties [25].

The study aimed to explore new MOFs with distinctive framework structures by desymmetrization of the bridging linkers. For example, the ditopic 3,3′-terphenyldicarboxylate (3,3′-TPDC^2−^) with *C*_2h_ point group symmetry hindered the complete coordination of the auxiliary ditopic N-donor pillar ligand (1,4-diazabicyclo [2.2.2]octane, DABCO) and produced only singly coordinated DABCO ligands inside the MOF channel [26,27]. In comparison, dicarboxylate linkers with more symmetric *D*_2h_ point group symmetry mostly led to doubly coordinated DABCO pillar ligands [19,28,29,30]. Jiang et al., also demonstrated the linker desymmetrization strategy to prepare a highly stable porphyrin-MOF named USTC-9. The stable USTC-9 showed the same topology as PCN-600, which easily lost its crystalline structure upon conventional activation [31]. Xie et al., also prepared three new MOFs using desymmetrized tetracarboxylate ligands [32]. Interestingly, these MOFs maintained the original topological structure of NOTT-115 prepared from a highly symmetric hexacarboxylate linker. Therefore, even slight desymmetrization of the bridging ligand tends to dramatically affect the final framework structure, functionality, and stability. In this way, one can easily tailor the framework structure and its relevant properties. A plethora of 2D lamellar MOFs were also reported. Especially, these 2D lamellar MOFs could be frequently obtained when low-symmetry N-donor bridging ligands and dicarboxylate co-linkers were used. For example, 2D microporous MOFs produced through the simultaneous use of low-symmetry N-donor bridging ligands with carboxylate bridging linkers were previously reported [18].

In this study, a ditopic N-donor bridging ligand with a pyrene core, 1,6-di(pyridin-3-yl)pyrene (3-DPPy), which can potentially act as a pillar ligand with dicarboxylate co-linker during the formation of MOF, was prepared. 3-DPPy containing 3-positioned pyridyl groups is less symmetric than the analogous 1,6-di(pyridin-4-yl)pyrene (4-DPPy) linker. Therefore, by using 3-DPPy with a dicarboxylate co-linker, the study successfully prepared a new 2D lamellar Zn-MOF, [Zn(μ-3-DPPy)(μ-BDC)]_n_ (**1**). The crystal structure of Zn-MOF was analyzed by X-ray crystallography and its fluorescent properties were investigated in the solid state. The catalytic activities of Zn-MOF for the transesterification of esters were also investigated.

## 2. Results and Discussion

### 2.1. Preparation of the Bridging Linker and Zn-MOF

**Figure 1:**
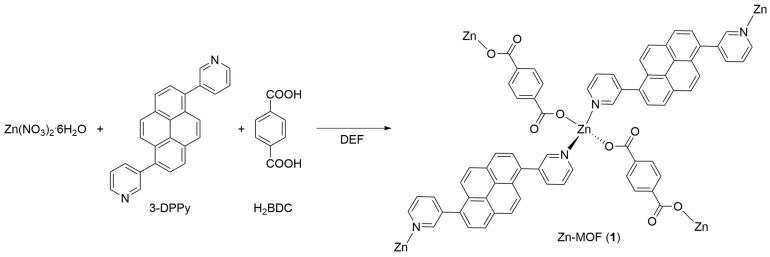
Schematic illustration of the formation of 2D lamellar Zn-MOF (**1**) from Zn(II) ions, 3-DPPy, and H_2_BDC bridging linkers in *N*,*N*-diethylformamide (DEF).

The ditopic N-donor ligand with a pyrene core, 1,6-di(pyridin-3-yl)pyrene (3-DPPy), was prepared according to the method described in the literature [33,34] through the Suzuki coupling reaction between 1,6-dibromopyrene and 3-pyridinylboronic acid. The purity of the final solid product was unambiguously confirmed by ^1^H NMR spectroscopy. The title Zn-MOF was prepared by heating a mixture of Zn(NO_3_)_2_·6H_2_O, 3-DPPy, and 1,4-benzenedicarboxylic acid (H_2_BDC) under autogenous pressure in a high-pressure reactor, as illustrated in Figure 1. The resulting colorless block crystals were high quality and suitable for X-ray structure determination (Figure S1). Elemental analysis of the as-prepared Zn-MOF suggested the chemical formula of [Zn(μ-3-DPPy)(μ-BDC)]_n_ (**1**).

### 2.2. Crystal Structure and Topology Analysis of Zn-MOF

**Figure 2:**
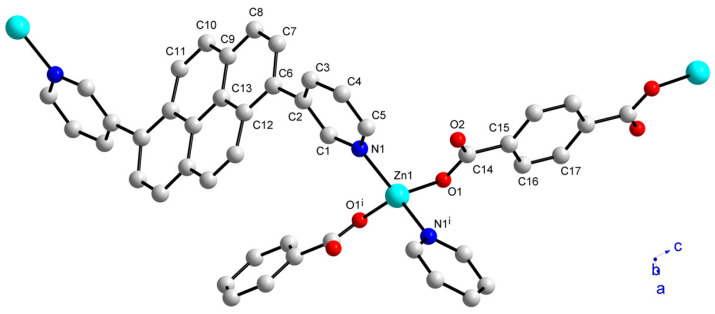
The asymmetric unit indicating the coordination environment of the Zn(II) ion in **1**. The labeled atoms represent the asymmetric unit (symmetry code: (i) 2-x, y, 3/2-z). Hydrogen atoms are omitted for clarity.

To further confirm the structure of **1**, a single crystal X-ray diffraction study was performed. The crystal structure indicates that a distorted tetrahedral Zn(II) ion is coordinated by two N atoms from two 3-DPPy linkers and two O atoms from BDC^2−^ linkers, as depicted in Figure 2. The bond angles are 117.6(1), 122.63(14), and 96.69(13)° for N1-Zn1-O1, N1-Zn1-N1^i^, and O1-Zn1-O1^i^, respectively (symmetry code: (i) 2-x, y, 3/2-z). Interestingly, the O atoms only show η^1^-coordination mode without forming a bidentate chelate bond. As a result, the frequently observed dinuclear Zn_2_(COO)_4_ paddlewheel SBU was not formed in this case. We speculate that the low symmetric positions of the two pyridyl N-donor atoms in 3-DPPy may prevent the formation of more common Zn_2_(COO)_4_ paddlewheel SBU or other cluster-based SBU structures. The study observed that the less symmetric ditopic bridging linkers often produced distinctive framework structures compared to the structures with more symmetric linkers [26,35]. Thus, the desymmetrization of bridging linkers is a good method for developing new MOF structures.

**Figure 3:**
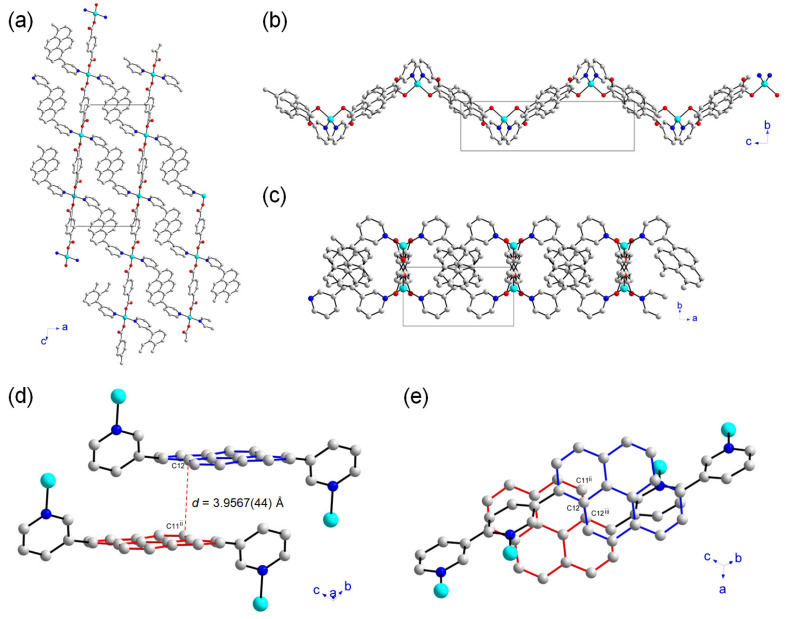
The crystal structure of a single sheet of **1** along the *b*-axis (**a**), *a*-axis (**b**), and *c*-axis (**c**). The unit cell is indicated as a black color box. (**d**) The side view of the two neighboring pyrene rings, indicated in different colors, shows the interplanar distance estimated from the two closest carbon atoms, C12 and C11^ii^ (symmetry code: (ii) x, -1+y, z). (**e**) Top view of the two neighboring pyrene rings projected onto the molecular planes showing their relative orientations (symmetry codes: (ii) x, -1+y, z and (iii) 1-x, 1-y, 1-z). Hydrogen atoms are omitted for clarity.

The framework structure of **1** is an infinite 2D sheet, and these sheets tightly pack to form the lamellar structure, as shown in Figure 3. Each sheet is not planar but has an undulated wavy geometry, as depicted in Figure 3b. As clearly seen in Figure 2, the dihedral angle between the pyrene moiety and each substituted pyridyl group is quite large, and they show a rotated conformation based on the dihedral angle of 52.913(442)° for C7-C6-C2-C3 bonds. This configuration, as well as the η^1^-coordination mode of the carboxylate groups, led to a new 2D **1**. The topology of the **1** was further investigated by ToposPro [36]. It showed a 2D **sql** network when each Zn(II) ion was considered as a node (Table S1). The term **sql** is the abbreviated form of square lattice topology in the Reticular Chemistry Structure Resource (RCSR). Many different types of inorganic crystal structures with simple nets are systematically indicated by the RCSR abbreviations available at http://rcsr.net accessed on 15 July 2023. The Schläfli symbol of this 4-connected unimodal net is {4^4^·6^2^}. The closest distance between the pyrene rings is an important parameter for governing the fluorescence properties of the compound containing pyrene moieties. The interplanar distance between two neighboring pyrene moieties for **1** was estimated to be 3.9567(44) Å, as shown in Figure 3d. Although the two neighboring pyrene rings orient in a face-to-face fashion, they are not effectively overlapped, as depicted in Figure 3e. Thus, two pyrene rings are showing a parallelly displaced stacking. These structural features may strongly affect the fluorescence properties of **1**. In comparison, two neighboring pyrene molecules without any substituents showed very efficient overlapping π-π stacking interactions in a face-to-face fashion to form an excimer [37]. The interplanar distance between two pyrene molecules is about 3.5 Å. This type of stacking of aromatic dipoles tends to cancel the net dipoles [38].

**Figure 4:**
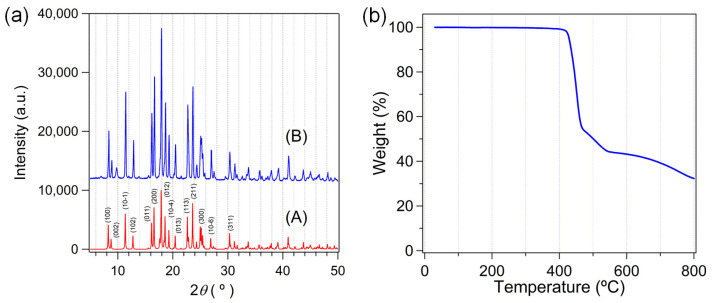
(**a**) The PXRD patterns of the simulated data (A) and as-prepared **1** (B). Representative Miller indices are shown for A. (**b**) The TGA curve for as-prepared **1**.

The powder X-ray diffraction (PXRD) pattern of as-prepared **1** is perfectly matched with the simulated pattern from single crystal X-ray data, as shown in Figure 4a. Thus, the bulk purity of the as-prepared sample is very high. Thermogravimetric analysis (TGA) also indicated the possible formation of **1**. There is almost no weight loss upon a temperature increase of up to 400 °C, as shown in Figure 4b. This TGA result implies that **1** does not contain any volatile solvate molecules or aqua ligands which can be easily removed at an elevated temperature. Both PXRD and TGA results agree well with the crystallographically determined structure of **1**.

### 2.3. Fluorescent Properties of Zn-MOF and 3-DPPy in the Solid State

Since the pyrene moiety of the ditopic bridging 3-DPPy linker is a well-known fluorophore, the fluorescent properties of 3-DPPy and **1** were investigated. Pyrene can form an excimer due to π-π stacking interactions when the distance between pyrene molecules is about 3.5 Å [37,39], and a broad featureless excimer emission band is usually observed at 470–480 nm [40,41]. The excimer emission is observable when the excited pyrene (pyrene *) and ground state pyrene are in close proximity. The excimers start to form at concentrations above ca. 10 μM in solution (Figure S2). In contrast, multiple monomer emission bands were observed at 375–400 nm [42,43]. Thus, both emission modes are easily distinguishable because of their different wavelengths. Marder et al. also reported the fluorescence properties of 4-DPPy and other pyrene derivatives with varying pyridyl substituents at different substitutional positions of the pyrene core [22].

**Figure 5:**
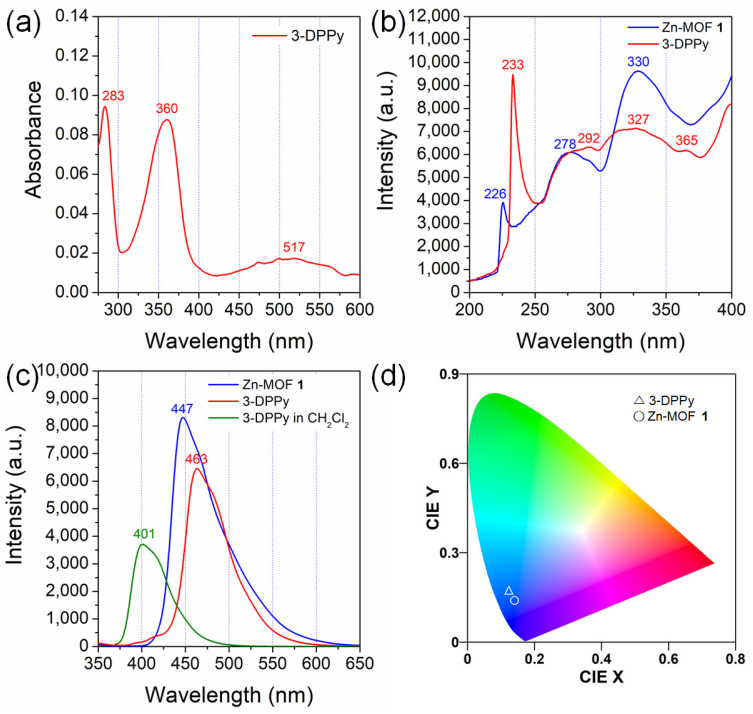
(**a**) UV-Vis absorption spectrum of 3-DPPy in CH_2_Cl_2_ (*C* = 10 μM). (**b**) Solid-state excitation spectra of **1** (λ_em_ = 447 nm) and 3-DPPy (λ_em_ = 463 nm). (**c**) Solid-state emission spectra of **1** and 3-DPPy (λ_ex_ = 343 nm) and the emission spectrum of 3-DPPy dissolved in CH_2_Cl_2_ (*C* = 10 μM, λ_ex_ = 360 nm). (**d**) The CIE color coordinate diagram for **1** and 3-DPPy.

Generally, the unsubstituted pyrene monomer showed four main absorption transitions in cyclohexane [41]: 243 (S_4_ ← S_0_ transition), 272 (S_3_ ← S_0_ transition), 334 (S_2_ ← S_0_ transition), and 372 nm (S_1_ ← S_0_ transition). Among these four transitions, the signal corresponding to the S_1_ ← S_0_ transition is exceptionally weak in the solution. These known data were used for comparison with those of 3-DPPy and **1,** despite the presence of pyridyl substituents. The study first analyzed the solution absorption behavior of 3-DPPy. The UV-Vis spectrum of the low-concentration solution of 3-DPPy (10 μM in dichloromethane) shows three absorption bands at 283, 360, and 517 nm, as shown in Figure 5a. The absorption maxima at 360 nm are thought to mainly be the fluorescence excitation signal for the monomeric pyrene emission [41]. This absorption band position is very close to that of 4-DPPy (358 nm) in the same concentration of toluene solution [22]. The very broad absorption band with a low intensity at around 517 nm is attributable to intermolecular aromatic interactions between 3-DPPy molecules [38]. Meanwhile, the solid-state fluorescence excitation spectrum of 3-DPPy showed four main excitation bands: 233, 292, 327, and 365 nm, as shown in Figure 5b. Considering the data for unsubstituted pyrene, these four excitation bands can be attributable to the S_4_ ← S_0_, S_3_ ← S_0_, S_2_ ← S_0_, and S_1_ ← S_0_ transitions, respectively. Contrarily, there are three main fluorescence excitation bands for **1** centered at 226, 278, and 330 nm. The relatively weak S_1_ ← S_0_ transition signal is not observed in this case.

The solid-state fluorescence spectra of 3-DPPy crystals and **1** are shown in Figure 5c. The normalized spectra are shown in Figure S3. The shapes of both bands are similar to each other, but 3-DPPy shows a more prominent shoulder at around 481 nm. Furthermore, their emission maxima are quite different. Overall, **1** shows a blue-shifted emission band at 447 nm compared to free 3-DPPy (463 nm). Most *d*^10^ Zn(II) complexes only show ligand-based fluorescence through π-π * transition because Zn(II) complexes usually do not have meaningful luminescent triplet charge-transfer-excited states unlike the isoelectronic Cu(I) complexes [44]. In Zn(II) complexes, the ligand-centered charge transfer (LCT) nature is mainly responsible for the lowest energy excited states for emissions [45]. Thus, less effective overlapping of the pyrene rings in **1** is likely to be one of the reasons for the large blue-shift compared to 3-DPPy crystals. The Commission Internationale d’Eclairage (CIE) coordinates of both samples were estimated by GoCIE software (ver. 2, Roorkee, India) using their emission data [46]. The CIE chromaticity coordinates of 3-DPPy (0.12, 0.17) changed into (0.14, 0.14) for **1,** as shown in Figure 5d. Interestingly, this CIE chromaticity coordinate for **1** indicates that **1** is an ideal blue light-emitting phosphor for light-emitting diodes (LEDs) [47,48,49].

Blue light-emitting phosphors can also be integrated into white light-emitting LED chips [50]. Recently, MOF-based blue light-emitting phosphors have attracted enormous attention because of their stability when compared to other materials [51]. Meanwhile, a recent example of the porphyrin-conjugated pyrene group acted as a good photosensitizer for CO_2_ reduction by the Fe-porphyrin catalytic center [52]. These examples demonstrate the importance of the blue light-emitting properties of pyrene moieties in materials chemistry.

The emission band for the unsubstituted pyrene crystals was seen at 470 nm [42]. The low-concentration solution of 3-DPPy shows a blue-shifted monomer emission band relative to the solid state. The fluorescence spectrum of 3-DPPy in CH_2_Cl_2_ shows a broad single emission band at 401 nm, with a shoulder (Figure 5c). Interestingly, when comparing the fluorescence of **1** with 4-DPPy, the emission bands for 4-DPPy were observed at 339 (solution state) and 457 nm (solid state) [22]. Thus, **1** showed a higher emission energy than both 3-DPPy and 4-DPPy in the solid state. Furthermore, the crystal structure of 4-DPPy showed no π-π stacking interaction between the pyrene moieties [22]. Instead, there were weak C-H⋯N and C-H⋯π interactions. Therefore, the emission band at 457 nm for 4-DPPy is not from the well-defined excimers. The crystal structure clearly supports this behavior.

**Figure 6:**
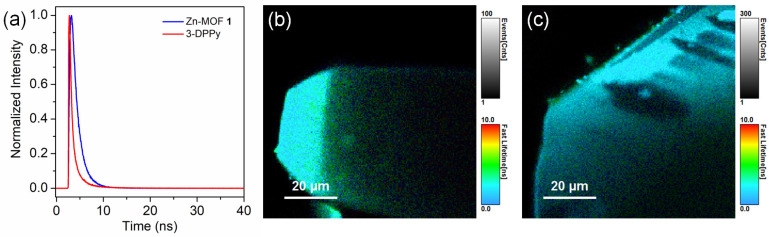
(**a**) Fluorescence decay curves for **1** and 3-DPPy (λ_ex_ = 375 nm). (**b**) Image from fluorescence lifetime imaging (FLIM) analysis of **1**. (**c**) FLIM image of 3-DPPy.

The fluorescence lifetimes of **1** and 3-DPPy were investigated by time-resolved photoluminescence (TRPL) measurements using a time-correlated single-photon counting technique at ambient conditions. The decay profiles are depicted in Figure 6a. The fluorescence of 3-DPPy decays slightly faster than **1**. The biexponentially fitted curve for 3-DPPy indicated lifetime values of τ_1_ = 1.29 ± 0.017 and τ_2_ = 2.55 ± 0.082 ns. For **1**, the triexponentially fitted curve indicated lifetime values of τ_1_ = 0.47, τ_2_ = 1.81, and τ_3_ = 9.7 ± 0.24 ns. The intensity average lifetimes are 1.51 ± 0.003 and 1.85 ± 0.022 ns for 3-DPPy and **1**, respectively. Figure 6b,c show the images from fluorescence lifetime imaging analysis and demonstrate the marginal difference between **1** and 3-DPPy. Thus, the formation of **1** containing 3-DPPy does not significantly change the fluorescence lifetime of 3-DPPy.

### 2.4. Catalytic Transesterification Activity of Zn-MOF

**Figure 7:**
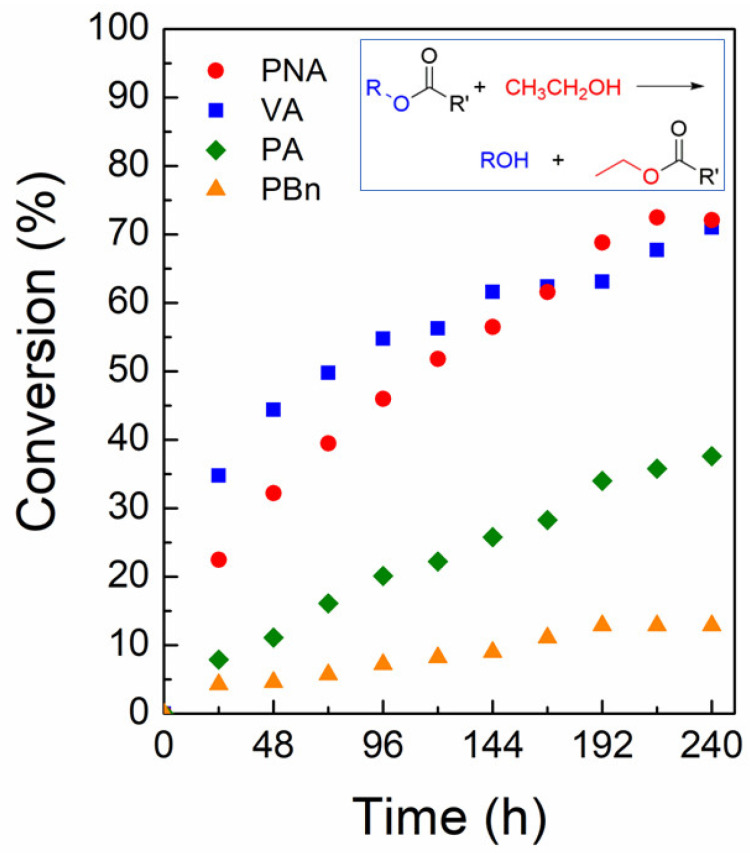
Time courses for the transesterification reaction of four different substrates catalyzed by **1**. The inset indicates the transesterification reaction between esters and ethanol to generate ethyl acetate or ethyl benzoate and the corresponding alcohols.

**Figure 8:**
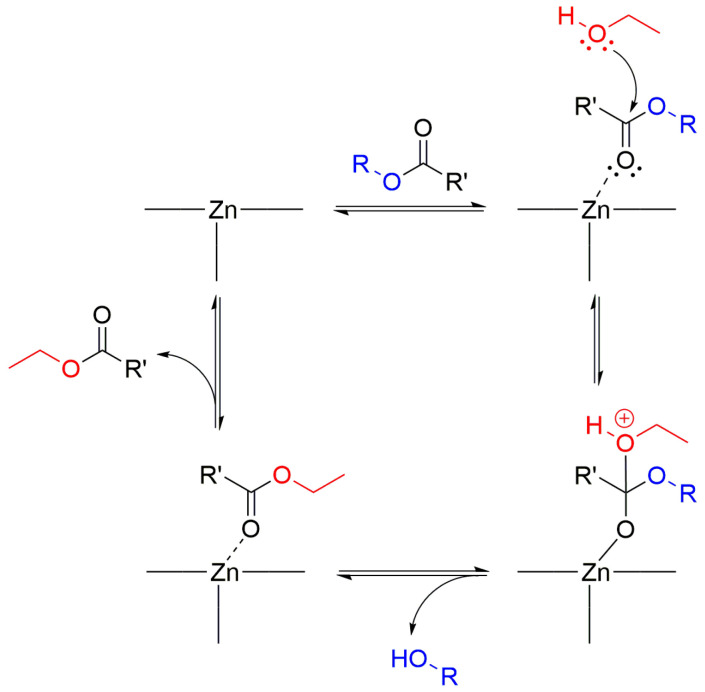
Proposed catalytic cycle for the transesterification of esters with ethanol by **1** with Zn(II) defect sites.

Although **1** is a nonporous 2D lamellar framework, its crystal surfaces contain many Zn(II) centers. There are defect sites among these surface Zn(II) centers that can act as Lewis acidic sites for catalysis [53]. Furthermore, the crystal habit of **1** is a block shape with a relatively thin thickness. Thus, it is beneficial to use these crystals as a nontoxic heterogeneous Lewis acid catalyst for organic transformations. The transesterification of esters was chosen as a model reaction due to its importance in the production of pharmaceuticals and biodiesel [53,54]. The transesterification reaction can be catalyzed by Lewis acid catalysts as well as Lewis base catalysts [55]. The study tested four representative ester substrates for transesterification with ethanol at 50 °C. The new ester products are ethyl acetate or ethyl benzoate and the corresponding alcohols. The substrates are 4-nitrophenyl acetate (NPA), vinyl acetate (VA), phenyl acetate (PA), and phenyl benzoate (PBn). The time-dependent conversion of each substrate is shown in Figure 7. The conversions for the desired product are 72 (NPA), 71 (VA), 38 (PA), and 13% (PBn). Generally, NPA shows a much faster conversion than the other substrates due to the favorable electronic effect of the nitro substituent. Interestingly, the smaller-sized VA displayed a very comparable rate to NPA, while the large PA and PBn showed low conversions. This reactivity order is similar to that observed previously for the same reaction catalyzed by the Cu-based 3D MOF of [{Cu_2_(glu)_2_(μ-bpa)}·(CH_3_CN)]_n_ (glu = glutarate, bpa = 1,2-bis(4-pyridyl)ethane) in methanol at 50 °C [17]. Although this 3D Cu-MOF is microporous, the catalytic reaction may mostly occur on its surfaces because its pore dimension (5.37 × 4.09 Å) is relatively small. Contrarily, the microporous 2D Zn-based [Zn(glu)(µ-bpe)]·2H_2_O (bpe = 1,2-bis(4-pyridyl)ethylene) MOF exhibited a much lower conversion for VA compared to NPA [18]. In this case, NPA showed about 6.6-fold enhanced conversion relative to VA. A Zn-bisSalen MOF with a Lewis basic pyridyl group inside the micropores has also shown a 2.5-fold larger conversion of NPA over VA [56]. These results indicate that **1** is a good transesterification catalyst, especially for NPA and VA. The remarkable catalytic activity for VA is mainly attributable to its small molecular size because small substrates can easily be accessible to the surface reaction sites. In contrast, the largest PBn substrate showed quite low conversion compared to both VA and PA. The proposed reaction mechanism, which is analogous to the previously proposed mechanism for the homogeneous Lewis acidic Sn-based catalyst, is shown in Figure 8 [57].

## 3. Materials and Methods

### 3.1. Preparation of 3-DPPy

The 1,6-di(pyridin-3-yl)pyrene (3-DPPy) was prepared according to the method described in the literature [34]. The reaction mixture containing 1,6-dibromopyrene (0.7208 g, 2.0 mmol, TCI) and 3-pyridinylboronic acid (0.7382 g, 6.0 mmol, TCI) in a mixed solvent of tetrahydrofuran (28 mL), toluene (4 mL), and 2.0 M aqueous K_2_CO_3_ (24 mL) was deoxygenated by the bubbling of dry N_2_ gas for 2 h. Next, Pd(PPh_3_)_4_ (0.0702 g, 0.061 mmol, TCI) was added to the reaction mixture. The mixture was then heated under reflux with constant stirring in an N_2_ atmosphere for 60 h. The suspension was cooled to room temperature and evaporated to dryness using a rotary evaporator. The dark solids were dissolved in a mixture of dichloromethane (100 mL) and distilled water (100 mL), and the oil phase was separated. This extraction process was repeated three times. The final oil phase was dried over anhydrous Na_2_SO_4_. The Na_2_SO_4_ was removed by filtration and the filtrate was evaporated to dryness by a rotary evaporator. The solids were purified by SiO_2_ chromatography using an eluent of dichloromethane and methanol (95:5 *v*/*v*%). The product band was collected, and the solvent was removed in vacuo. The solids were further purified by recrystallization in hot toluene to give a yield of 0.536 g (75%). The purity of the final solid product was confirmed by ^1^H NMR spectroscopy. ^1^H NMR (CDCl_3_, 400.13 MHz, *δ*): 8.91 (*d*, 2H, 2.0 Hz), 8.76 (*dd*, 2H, 4.8, 1.6 Hz), 8.27 (*d*, 2H, 8.0 Hz), 8.12 (*m*, 4H), 7.98 (*m*, 4H), 7.53 (*dd*, 2H, 7.6, 4.8 Hz).

### 3.2. Preparation of Zn-MOF ***1***

The reaction mixture consisting of Zn(NO_3_)_2_·6H_2_O (0.0594 g, 0.2 mmol, Merck, St. Louis, MO, USA), 3-DPPy (0.0712 g, 0.2 mmol), and 1,4-benzenedicarboxylic acid (0.0332 g, 0.2 mmol, Merck, St. Louis, USA) in 20 mL of *N*,*N*-diethylformamide (DEF, TCI, Fukaya City, Japan) was sealed in a high-pressure reactor (100 mL) and heated at 120 °C under autogenous pressure for 3 d. The reactor was slowly cooled down to room temperature. The resulting colorless crystals were obtained by filtration and washed with DEF. The crystals were air-dried for a few days to give a yield of 0.0583 g (50%). Anal. calcd. for C_34_H_20_N_2_O_4_Zn (585.92): C 69.70, H 3.44, N 4.78; found: C 69.40, H 3.49, N 4.87%.

### 3.3. Instrumentation

NMR spectra were recorded using a Bruker Ascend 400 (400.13 MHz for ^1^H) spectrometer. The proton chemical shifts of the samples were calibrated with respect to the reference proton resonance signals occurring from the protic residues of the deuterated solvents. The elemental composition of the MOF sample was analyzed at the Organic Chemistry Research Center (Seoul, Republic of Korea). Thermogravimetric analysis was carried out using a TGA Q5000 (TA Instruments, New Castle, DE, USA) under a nitrogen atmosphere (ramping rate = 15 °C min^−1^). PXRD spectra were recorded on a Bruker D8 Focus diffractometer (40 kV, 30 mA, step size = 0.02°). UV-Vis spectra were collected by a Scinco S-3100 spectrophotometer. Solid-state fluorescence spectra were collected using a Hitachi F-4500 fluorescence spectrophotometer. Gas chromatographic analysis was performed using a HP-5890 Series II gas chromatograph equipped with a flame ionization detector (FID) and a capillary column (Supelco SPB-1; L × I.D. 30 m × 0.25 mm, df 1.00 μm). The retention times of the desired products of transesterification were determined by using commercially available authentic samples, and n-nonane was used as an internal standard for quantification.

### 3.4. Photoluminescence Lifetime Measurements

Time-resolved photoluminescence (TRPL) imaging was performed using an inverted-type scanning confocal microscope (MicroTime-200, Picoquant, Germany) with a 60× (water) objective. The measurements were performed at the Korea Basic Science Institute, Daegu Center. A single-mode pulsed diode laser (470 nm with ~30 ps pulse width and an average power of <1 μW) was used as an excitation source. A dichroic mirror (490 DCXR, AHF), a long-pass filter (HQ500lp, AHF), and a single-photon avalanche diode (SPAD; PDM series, MPD) were used to collect the emission data from each sample. A time-correlated single-photon counting technique was used to count emitting photons. TRPL images with a dimension of 80 × 80 μm^2^, which consisted of 200 × 200 pixels, were recorded using a time-tagged time-resolved (TTTR) data acquisition route. The acquisition time was 2 ms for each pixel. Exponential fittings for the obtained PL decays were performed using the SymPhoTime-64 software (ver. 2.7, Berlin, Germany).

### 3.5. Crystal Structure Determination of Zn-MOF ***1***

The X-ray intensity data of **1** were measured on a Bruker APEX-II diffractometer equipped with a monochromator and a Mo Kα X-ray source (λ = 0.71073 Å). The crystal was mounted on the loop for X-ray data collection at 223 K. The Bruker-SAINT software package (ver. 6.45, Madison, WI, USA) was used for the integration and scaling of the collected CCD data. The crystal structures were solved and refined using the ShelXle suite [58]. All hydrogen atoms were placed in their calculated positions. The crystal data and refinement result are shown in Table S2. Bond distances and bond angles are given in Table S3. Structural information was deposited at the Cambridge Crystallographic Data Center (CCDC 2268972).

### 3.6. Transesterification of Esters by Zn-MOF ***1***

A mixture of the as-prepared **1** catalyst (0.004 g, 0.0069 mmol), an ester of choice (0.279 mmol (4-nitrophenyl acetate, 0.051 g; phenyl acetate, 0.038 g; vinyl acetate, 0.024 g; phenyl benzoate, 0.055 g)), and n-nonane (10 μL, 0.056 mmol) as an internal standard in ethanol (10 mL) was heated at 50 °C with constant stirring for 240 h. The reaction progress was monitored at regular intervals by taking an aliquot and analyzing it by gas chromatography.

## 4. Conclusions

A new 2D lamellar framework of [Zn(μ-3-DPPy)(μ-BDC)]_n_ (**1**) was prepared and crystallographically characterized by X-ray diffraction. The desymmetrization of the bridging linker (3-DPPy) successfully produced a new Zn(II)-based 2D lamellar structure with a **sql** network. The 3-DPPy bridging linker prevented the formation of a dinuclear paddle-wheel type of SBU, and therefore a mononuclear Zn(II) center with a distorted tetrahedral geometry was obtained. Each undulated sheet packs together to form flat block crystals. The solid-state fluorescence of **1** was also investigated and the blue light-emitting properties, mainly from the 3-DPPy bridging linker, were confirmed. The framework of **1** showed slightly blue-shifted emission compared to the free 3-DPPy, based on emission spectra and corresponding CIE chromaticity coordinates. Interestingly, **1** also exhibited catalytic activity for the transesterification of esters, possibly because of the Zn(II) defect sites on the surfaces. In addition, **1** effectively catalyzed both NPA and VA compared to the PA and PBn substrates.

## Data Availability

Not applicable.

## Supplementary Materials

The following supporting information can be downloaded at: https://www.mdpi.com/article/10.3390/molecules28176357/s1, Figure S1: microscopic image of Zn-MOF **1**; Figure S2: normalized emission spectra of pyrene dissolved in ethanol (λ_ex_ = 336 nm, air-equilibrated condition); Figure S3: normalized solid-state emission spectra of **1** and 3-DPPy (λ_ex_ = 343 nm), and the emission spectrum of 3-DPPy dissolved in CH_2_Cl_2_ (*C* = 10 μM, λ_ex_ = 360 nm); Table S1: ToposPro analysis result; Table S2: crystal data and structure refinement for **1** (CCDC 2268972); Table S3: the bond distances (Å) and angles (°) for **1** (Symmetry codes: a = 2-x, y, 3/2-z; b = 1-x, 2-y, 1-z; c = 2-x, 2-y, 2-z).

## Sample Availability

Samples of the compounds are available from the authors.
