# Supplementary material for: Fluorescent and Catalytic Properties of a 2D Lamellar Zn Metal–Organic Framework with sql Network Structure
Source: Molecules. 2023 Aug 30;28(17):6357. doi: 10.3390/molecules28176357 (PMC10488886; doi:10.3390/molecules28176357)
Supplement: Supplementary file 1 [file molecules-28-06357-s001.zip › molecules-2573275-supplementary.pdf]

Supporting Information

Fluorescent and Catalytic Properties of a 2D Lamellar Zn Metal–Organic Framework with sql Network Structure

Chaewon Shin †, Jongseo Kim † and Seong Huh *

Department of Chemistry and Protein Research Center for Bio-Industry, Hankuk University of Foreign Studies, Yongin 17035, Republic of Korea; amyshin1007@naver.com (C.S.); rlawhdtj1020@naver.com (J.K.)

* Correspondence: shuh@hufs.ac.kr; Tel.: +82-31-330-4522

† These authors contributed equally to this work.

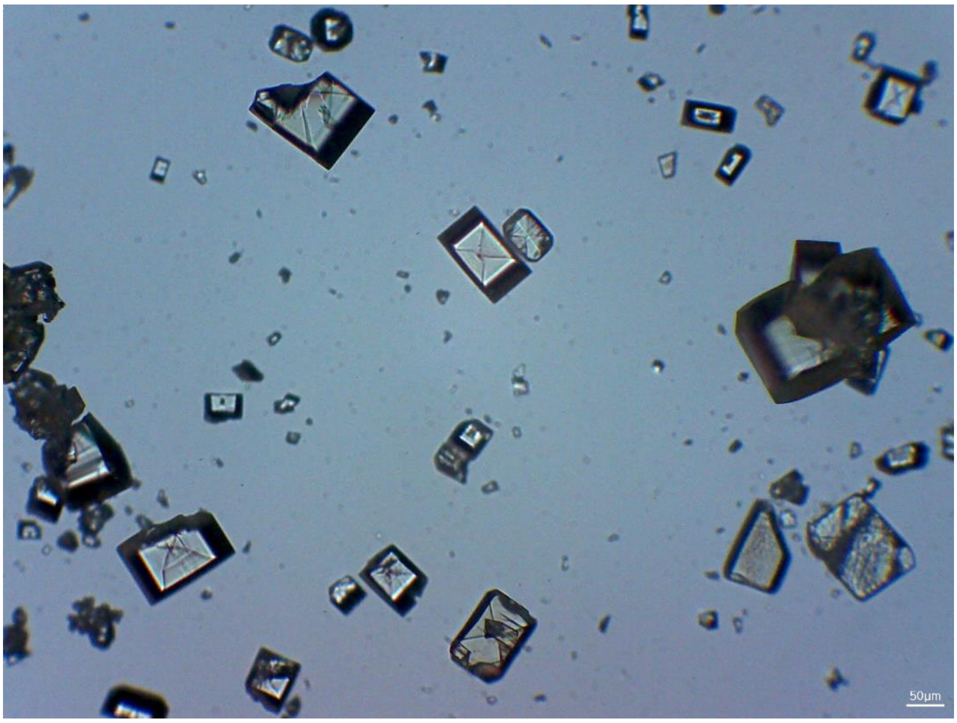

Figure S1. Microscopic image of Zn-MOF **1**.

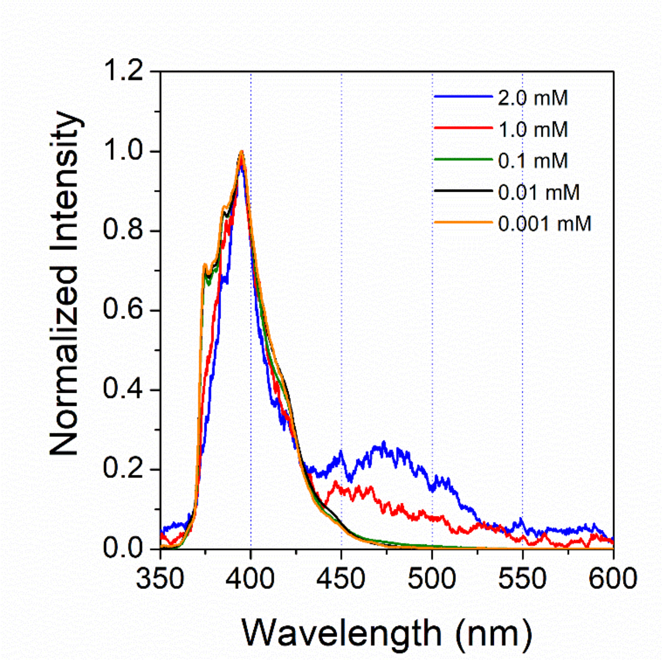

Figure S2. Normalized emission spectra of pyrene dissolved in ethanol (λ_{ex} = 336 nm, air-equilibrated condition).

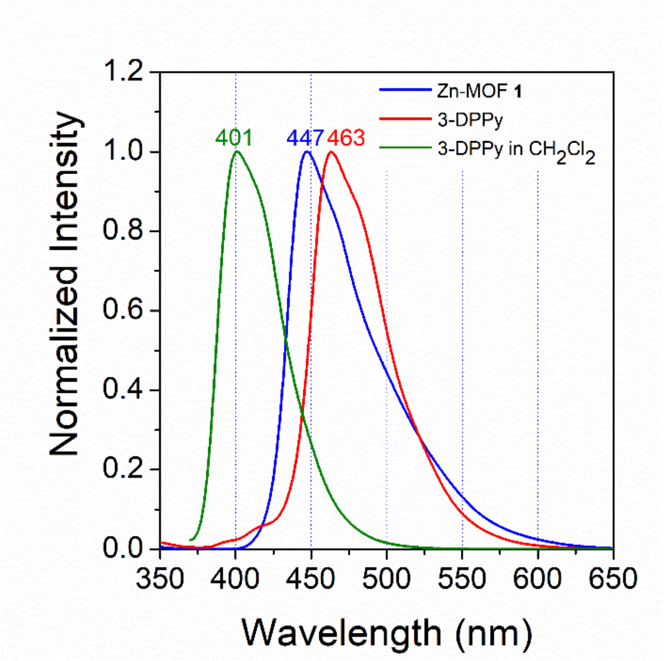

Figure S3. Normalized solid-state emission spectra of **1** and 3-DPPy (λ_{ex} = 343 nm), and the emission spectrum of 3-DPPy dissolved in CH_2Cl_2 (C = 10 μM, λ_{ex} = 360 nm).

Table S1. ToposPro analysis result.

##################
1:C34 H20 N2 O4 Zn
##################

Topology for Zn1

Atom Zn1 links by bridge ligands and has

Common vertex with					R(A-A)	f
Zn 1	0.0000	1.3609	0.2500	(0 2 1)	10.941A	1
Zn 1	0.0000	1.3609	1.2500	(0 2 2)	10.941A	1
Zn 1	1.0000	1.3609	1.2500	(1 2 2)	14.501A	1
Zn 1	-1.0000	1.3609	0.2500	(-1 2 1)	14.501A	1

Structural group analysis

Structural group No 1

Structure consists of layers (0 1 0) with ZnO4N2C34H20
Num. groups=1; Thickness=6.89; Distances to Neighbors=5.700; 5.700

Coordination sequences

Zn1:	1	2	3	4	5	6	7	8	9	10
Num	4	8	12	16	20	24	28	32	36	40
Cum	5	13	25	41	61	85	113	145	181	221

TD10=221

Vertex symbols for selected sublattice

Zn1 Point symbol:{4^4.6^2}
Extended point symbol:[4.4.4.4.6(2).6(2)]

Point symbol for net: {4^4.6^2}
4-c net; uninodal net

Topological type: sql (topos&RCSR.ttd) {4^4.6^2} - VS [4.4.4.4.*.*] (17892 types in 4 databases)
Elapsed time: 7.56 sec.

Table S2. Crystal data and structure refinement for **1** (CCDC 2268972).

Empirical formula	$C_{34}H_{20}N_2O_4Zn$
Formula weight	585.91
T (K)	223
Color	colorless
Crystal size (mm^3)	0.06 × 0.07 × 0.08
Crystal system	Monoclinic
Space group	P2/c (13)
Wavelength (Å)	0.71073
a (Å)	10.759(3)
b (Å)	5.6999(15)
c (Å)	20.276(5)
α (°)	90
β (°)	96.627(9)
γ (°)	90
V (Å^3)	1235.1(6)
Z	2
ρ_{calcd} (g cm^{-3})	1.576
μ (mm^{-1})	1.042
F(000)	600
Reflections collected	8336
Independent reflections	2984 [R_{int} = 0.056]
Data / restraints / parameters	2984 / 0 / 186
GOF on F^2	1.07
Final R indices [I>2σ(I)][a,b]	R_1 = 0.0477, wR_2 = 0.1144
R indices (all data)[a,b]	R_1 = 0.0744, wR_2 = 0.1306
Largest diff. peak and hole (e Å^{-3})	0.52 and -0.38

[a] $R_1 = \Sigma||F_o| - |F_c||/\Sigma|F_o|$. [b] $wR_2 = [\Sigma w(F_o^2 - F_c^2)^2/\Sigma w(F_o^2)^2]^{1/2}$.

Table S3. The bond distances (Å) and angles (°) for **1** (Symmetry codes: a = 2-x,y,3/2-z; b = 1-x,2-y,1-z; c = 2-x,2-y,2-z).

Zn1-O1	1.980(2)
Zn1-O2	2.674(2)
Zn1-N1	2.043(2)
Zn1-O1a	1.980(2)
Zn1-O2a	2.674(2)
Zn1-N1a	2.043(2)
O1-C14	1.288(4)
O2-C14	1.234(4)
N1-C1	1.347(4)
N1-C5	1.347(4)
C1-C2	1.393(5)
C2-C3	1.392(4)
C2-C6	1.484(4)
C3-C4	1.387(5)
C4-C5	1.372(5)
C6-C7	1.407(5)
C6-C12	1.404(4)
C7-C8	1.378(5)
C8-C9	1.390(5)
C9-C10	1.437(5)
C9-C13	1.429(4)
C10-C11	1.348(5)
C11-C12b	1.445(5)
C12-C13	1.439(4)
C13-C13b	1.417(4)
C14-C15	1.510(4)
C15-C16	1.390(4)
C15-C17c	1.388(4)

C16-C17	1.384(5)
O1-Zn1-O2	54.47(8)
O1-Zn1-N1	117.59(9)
O1-Zn1-O1a	96.68(10)
O1-Zn1-O2a	146.34(8)
O1-Zn1-N1a	100.08(9)
O2-Zn1-N1	82.31(8)
O2-Zn1-O1a	146.34(8)
O2-Zn1-O2a	158.22(7)
O2-Zn1-N1a	87.27(8)
N1-Zn1-O1a	100.08(9)
N1-Zn1-O2a	87.27(8)
N1-Zn1-N1a	122.63(9)
O1a-Zn1-O2a	54.47(8)
O1a-Zn1-N1a	117.59(9)
O2a-Zn1-N1a	82.31(8)
Zn1-O1-C14	106.4(2)
Zn1-O2-C14	75.51(18)
Zn1-N1-C1	116.93(19)
Zn1-N1-C5	124.7(2)
C1-N1-C5	118.1(2)
N1-C1-C2	124.3(3)
C1-C2-C3	116.3(3)
C1-C2-C6	119.5(3)
C3-C2-C6	124.1(3)
C2-C3-C4	119.6(3)
C3-C4-C5	120.3(3)
N1-C5-C4	121.4(3)
C2-C6-C7	117.7(3)
C2-C6-C12	122.6(3)
C7-C6-C12	119.5(3)

C6-C7-C8	120.9(3)
C7-C8-C9	121.6(3)
C8-C9-C10	122.9(3)
C8-C9-C13	118.9(3)
C10-C9-C13	118.2(3)
C9-C10-C11	121.6(3)
C10-C11-C12b	121.9(3)
C6-C12-C13	119.4(3)
C6-C12-C11b	123.1(3)
C13-C12-C11b	117.5(3)
C9-C13-C12	119.4(3)
C9-C13-C13b	120.3(3)
C12-C13-C13b	120.3(3)
O1-C14-O2	123.1(3)
O1-C14-C15	116.4(3)
O2-C14-C15	120.5(3)
C14-C15-C16	121.0(3)
C14-C15-C17c	119.5(3)
C16-C15-C17c	119.5(3)
C15-C16-C17	120.6(3)
C16-C17-C15c	119.9(3)
